# *In Vitro* Assessment of the Probiotic Potential of *Lactococcus lactis* LMG 7930 against Ruminant Mastitis-Causing Pathogens

**DOI:** 10.1371/journal.pone.0169543

**Published:** 2017-01-09

**Authors:** Federica Armas, Cristina Camperio, Cinzia Marianelli

**Affiliations:** 1 Department of Food Safety and Veterinary Public Health, Istituto Superiore di Sanità, Rome, Italy; 2 Department of Sciences, Roma Tre University, Rome, Italy; 3 Department of Animal Pathology, Faculty of Veterinary Medicine, University of Turin, Turin, Italy; Universite Paris-Sud, FRANCE

## Abstract

Mastitis in dairy ruminants is considered to be the most expensive disease to farmers worldwide. Recently, the intramammary infusion of lactic acid bacteria has emerged as a potential new alternative to antibiotics for preventing and treating bovine mastitis. In this study we have investigated *in vitro* the probiotic potential of *Lactococcus lactis* LMG 7930, a food-grade and nisin-producing strain, against mastitis-causing pathogens. We have characterized its carbohydrate fermentation and antibiotic susceptibility profiles, cell surface properties and antimicrobial activity, as well as its capabilities to adhere to and inhibit the invasion of pathogens into the bovine mammary epithelial cell line BME-UV1d. We found that *L*. *lactis* LMG 7930 was sensitive to tested drugs, according to the EFSA Panel on Additives and Products or Substances used in Animal Feed (FEEDAP), and showed an improved carbohydrate fermentation capacity compared to starter strains. Moreover, the strain exhibited antagonistic properties towards many of the pathogens tested. It presented medium surface hydrophobicity, a low basic property and no electron acceptor capability. It showed low auto-aggregation and no co-aggregation abilities towards any of the tested pathogens. The strain was one of the most adhesive to bovine mammary epithelial cells among tested bacteria, but its internalisation was low. The strain did not affect significantly pathogen invasion; however, a trend to decrease internalization of some pathogens tested was observed. In conclusion, our results suggest that this strain might be a promising candidate for the development of new strategies of mastitis control in ruminants. Future investigations are needed to evaluate its safety and efficacy under field conditions.

## Introduction

Mastitis is an inflammation of the mammary gland that affects all mammals, especially ruminants in dairy farms. It is considered to be the most expensive disease to farmers worldwide due to the reduction in milk quantity and quality, and to animal treatment and replacement costs [1].

The main management strategies for preventing and treating mastitis in ruminants involve the extensive use of antibiotics that are often ineffective, especially regarding *Staphylococcus aureus* infections [2]. In addition, this widespread use of drugs increases the risk of antibiotic residues in milk and dairy products and the risk of transmission of antibiotic resistance to both commensal bacteria and opportunistic pathogens. The development of antibiotic alternatives, especially in the veterinary field, is therefore strongly advocated.

The use of lactic acid bacteria (LAB), generally recognised as safe, and their antimicrobial peptides (bacteriocins) has recently been proposed for the control of bovine mastitis. Nisin, currently the only bacteriocin widely used as a food preservative, exhibits antimicrobial activity against a wide range of Gram-positive bacteria, including foodborne and mastitis-causing pathogens. Formulations containing nisin administered to the bovine teat by dipping or intramammary infusion proved to be effective in restricting or treating staphylococcal and streptococcal infections [3–5]. Recently, *in vivo* studies on cattle revealed the probiotic potential of the intramammary infusion of live cultures of LAB against mastitis by eliciting a rapid mammary gland immune response [6–9]. However, this option remains poorly documented.

A number of *in vitro* studies have screened LAB strains for potential beneficial properties in order to select promising candidates to use *in vivo* for preventing and treating bovine mastitis [10–12]. Several *Lactococcus* and *Lactobacillus* strains have shown the ability to inhibit adhesion to and internalization in bovine mammary epithelial cells of mastitis-causing pathogens, as well as to modulate cell immune response [12–15], suggesting possible mechanisms to account for the positive results obtained *in vivo*.

In this study we have investigated *in vitro* the probiotic potential of *Lactococcus lactis* LMG 7930, a food-grade and nisin-producing strain, against mastitis-causing pathogens to evaluate its potential use as a new alternative in treating mastitis in ruminants. We have therefore characterized its carbohydrate fermentation and antibiotic susceptibility profiles, cell surface properties and antimicrobial activity, as well as its capabilities to adhere to and inhibit the invasion of pathogens into bovine mammary epithelial cells.

## Materials and Methods

### Bacterial strains and culture conditions

*L*. *lactis* subsp. *lactis* LMG 7930 (BCCM/LMG Bacteria Collection, Belgium), a nisin-producing strain used in the production of Swiss cheese to suppress gas production by Clostridia, was assessed *in vitro* for its probiotic potential.

Ten mastitis-causing pathogens, including two bovine reference mastitis strains from BCCM/LMG Bacteria Collection (Belgium) and eight mastitis field isolates from sheep with mastitis, were considered in our study and listed in Table 1. The mastitis field pathogens are from the bacteria collections of the Istituto Zooprofilattico Sperimentale della Sicilia and Istituto Zooprofilattico Sperimentale della Sardegna. The strains were previously isolated from milk of ewes with mastitis and characterized by biochemical and molecular (16S rDNA) tests.

**Table 1:** Mastitis-causing pathogens considered in the study.

Species	Strain	Host origin
*Staphylococcus aureus*	LMG 16805	bovine
*Streptococcus agalactiae*	LMG 14838	bovine
*Escherichia coli*	285–05	ovine
*Staphylococcus aureus*	357–08	ovine
*Staphylococcus chromogenes*	100-SA	ovine
*Staphylococcus epidermidis*	175–07	ovine
*Staphylococcus epidermidis*	200-SA	ovine
*Staphylococcus intermedius*	146–08	ovine
*Streptococcus agalactiae*	115–06	ovine
*Streptococcus dysgalactiae*	215–06	ovine

The nisin-sensitive *L*. *lactis* subsp. *cremoris* LMG 7951 (BCCM/LMG Bacteria Collection, Belgium) was used as positive control for antimicrobial activity testing.

Bacterial cultures were grown in brain heart infusion (BHI) broth (Oxoid, England) and incubated at 37°C for 24 h.

### Phenotypic characterization and antibiotic susceptibility testing of *L*. *lactis*

*L*. *lactis* was examined for its temperature tolerance at 30°C and 37°C. Its carbohydrate fermentation profile was also determined using API 50 CH assay (bioMérieux, France) according to the manufacturer’s instructions.

The antimicrobial susceptibility profile of *L*. *lactis* was determined for a total of 13 drugs: amikacin, ampicillin, ciprofloxacin, clarithromycin, chloramphenicol, erythromycin, gentamicin, kanamicin, linezolid, rifampicin, streptomycin, tetracycline and vancomycin. The broth microdilution method with Mueller Hinton medium (BD, USA) was used [16]. The test was carried out in triplicate in 96-well plates and based on the oxidation-reduction dye resazurin. For ampicillin (1–16 μg/ml), chloramphenicol (1–16 μg/ml), erythromycin (0.5–8 μg/ml), gentamicin (4–32 μg/ml), kanamicin (4–64 μg/ml), streptomycin (16–64 μg/ml), tetracycline (0.5–8 μg/ml), and vancomycin (0.5–8 μg/ml) the cut-off values identified by the EFSA Panel on Additives and Products or Substances used in Animal Feed (FEEDAP) [17] to distinguish between susceptible and resistant *L*. *lactis* strains were used. A bacterial strain is defined as resistant when it is not inhibited at a higher concentration of a specific antimicrobial than the established cut-off value. For the remaining drugs the following arbitrary MIC ranges were tested: amikacin (16–64 μg/ml), ciprofloxacin (1–16 μg/ml), clarithromycin (1–16 μg/ml), linezolid (1–8 μg/ml) and rifampicin (2–16 μg/ml). MICs were established as the lowest antibiotic concentration that inhibited bacterial growth.

### Surface characteristics

#### Adhesion to solvents

The bacterial adhesion to solvent (BATS) assay previously described [18] was used to determine *L*. *lactis* surface properties. Cell affinities for apolar and monopolar solvents which exhibit similar van der Waals surface tension components were investigated. The adhesion to xylene (apolar solvent) for surface hydrophobicity and the affinity to chloroform (polar acid solvent) and ethyl acetate (polar basic solvent) for the electron donor and electron acceptor properties, respectively, were assessed. Briefly, an overnight culture of *L*. *lactis* in BHI broth was centrifuged at 3000 x g for 5 min. The pellet was suspended in phosphate-buffered saline (PBS, pH 7.2; Sigma Aldrich Srl, Italy) to 10^8^ CFU/ml. A volume of this suspension (200 μl) was transferred into a microplate well and measured at 600 nm (A_0min_) using Bio Rad Model 550 Microplate Reader (Italy). An additional volume of the suspension (3 ml) was mixed with 1 ml of each solvent (xylene, chloroform or ethyl acetate). The three mixtures were vortexed for 1 min and allowed to stand for 5 min to separate into two phases. The aqueous phases were then measured with the spectrophotometer at 600 nm (A_5min_). The experiment was repeated twice. The mean absorbance values were calculated and affinities of *L*. *lactis* to solvents were expressed with the formula

$$BATS\;\left(\%\right)=\left(1-A_{5min}/A_{0min}\right)\;x\;100$$

#### Auto- and co-aggregation assays

The ability of *L*. *lactis* to self-aggregate and co-aggregate with pathogens was assessed as previously described [18,19]. Overnight bacterial cultures in BHI broth were centrifuged at 3000 x g for 5 min. Pellets were suspended in PBS pH 7.2 to 10^8^ CFU/ml.

For the auto-aggregation assay, 3 ml of *L*. *lactis* suspension was vortexed for 10 s, and absorbance was measured by the spectrophotometer at 600 nm (A_0h_). The suspension was incubated at 37°C for 2 h. The absorbance of the supernatant was then measured (A_2h_). The *L*. *lactis* self-aggregation ability was calculated using the formula

$$Auto-aggregation\;(\%)=\left(1-A_{2h}/A_{0h}\right)\;x\;100$$

For the co-aggregation assay, 1.5 ml of *L*. *lactis* suspension was mixed with 1.5 ml of each pathogen in separated tubes, vortexed for 10 s and incubated at 37°C for 2 h. The absorbance of each mixture was then measured at 600 nm (A_mix_) and compared to that of control tubes containing either *L*. *lactis* (A_*L*. *lactis*_) or the pathogen (A_pathogen_) at 2 h of incubation, following the formula

$$Co-aggregation\;(\%)=1-A_{mix}⁄[(A_{L.lactis}{}_{}+A_{pathogen})⁄2]\times 100$$

Adhesion to solvents and auto-aggregation and co-aggregation tests were performed twice. The mean values of absorbance were calculated and used in the formulas. Surface physicochemical properties and auto- and co-aggregation capabilities were classified as low, medium and high, according to their scores.

### Antimicrobial activity: Agar spot test

For detection of antagonistic activity of *L*. *lactis* towards mastitis-causing pathogens, the agar spot test previously described [20] was used on BHI agar plates. Briefly, overnight cultures of the target strains (pathogens) and the positive control (*L*. *cremoris*) were diluted in BHI broth, and 1 mL of ~10^6^ CFU/ml of each diluted culture was spread on BHI agar plates. After five min. of contact, the excess was removed and plates were left to dry for 10 min. Samples (3 μl) of a 24-h culture of *L*. *lactis* were spotted in triplicate on the agar surface. Plates were left to absorb and then incubated aerobically at 37°C. Inhibition zones were measured after 24 h of incubation. A clear zone of more than 1 mm around a spot was scored as positive. The test was performed twice. The mean halo radius values of triplicate spots from the two independent experiments were calculated.

### Adhesion and invasion assays

The bovine mammary epithelial cell line BME-UV1 was purchased from the Istituto Zooprofilattico Sperimentale della Lombardia e dell’Emilia Romagna (Brescia, Italy). BME-UV1 cells were cultured in a complete medium containing 40% of Ham’s F12 (Biowest), 30% of RPMI 1640 (Euroclone), 20% of NCTC 135 (Gibco), supplemented with 10% fetal calf serum (Euroclone), 0.1% of lactose (Fluka), 0.1% of lactoalbumin hydrolysate (Fluka), glutathione (1.2 mM, Fluka), L-ascorbic acid (10μg/ml, Sigma), hydrocortisone (1μg/ml, Sigma) and insulin (10μg/ml, Sigma). Cells were incubated at 37°C in 5% CO_2_, in a humidified atmosphere, to a confluent monolayer in tissue culture flasks (Corning, USA). For adhesion and invasion assays, cells were seeded in 96-well plates (3 x 10^4^ cells/well) and incubated overnight at 37°C in 5% CO_2_.

The adhesion and invasion abilities of both *L*. *lactis* and pathogens were assessed. Overnight bacterial cultures were centrifuged at 3000 x g for 5 min., and the pellet was washed in PBS solution and then suspended in cell growth medium to 10^8^ CFU/ml.

#### Adhesion assay

For the adhesion assessment each bacterial suspension (*L*. *lactis* or pathogen) was inoculated in triplicate to achieve a Ratio of Interaction (ROI; ratio of bacteria to cells) of 200:1. Microplates were incubated for 2 h at 37°C in 5% CO_2_. After incubation, supernatants were discarded, and the non-adherent bacteria were removed by washing each well twice with PBS. Monolayers were lysed by freeze-thawing twice in 100 μL of sterilized water per well. To enumerate the adhesion bacteria cell lysates were serially diluted (1:10) with PBS and pour-plated on BHI agar for *L*. *lactis*, MacConkey agar (Oxoid, England) for *E*. *coli*, Staphylococci 110 agar (Biolife Italiana, Italy) for staphylococcal strains and Trypticase Soy Agar with 5% Sheep Blood (TSA II; BD, New Jersey) for streptococcal strains with the exception of *S*. *agalactiae* LMG 14838 that was plated on CHROMagar Orientation (BD, New Jersey) because of its non-hemolytic property. All plates were incubated at 37°C for up to 48 h to allow the development of visible colonies. The mean population values across three plates per well were calculated. The medians and interquartile ranges of the triplicate samples were then determined.

#### Invasion assay

Invasion tests were performed as for adhesion assays with a bacterial ROI of 200:1 and an incubation time of 2 h. For invasion inhibition assay, *L*. *lactis* and each pathogen were simultaneously added to the cells at the same ROI and incubated for 2 h at 37°C. After incubation, monolayers were washed twice with PBS and incubated in cell growth medium containing gentamicin (100 μg/ml) for 1h at 37°C in order to kill extracellular bacteria. Monolayers were washed with PBS twice and then lysed. Subsequently, internalised bacteria were enumerated as described for adhesion assay. The cell invasion capability of each bacterium alone was also determined (control).

### Statistical analysis

Median and interquartile ranges were used to graphically describe inoculum, adhesion and invasion data sets. Differences between the internalization abilities of the pathogen populations alone (control) and those in the presence of *L*. *lactis*, were determined using the Mann-Whitney test. Differences were considered significant at *P*< 0.05. GraphPad Prism 6 version 6.07 for Windows, GraphPad Software, La Jolla California USA, www.graphpad.com, was used.

## Results

### Biochemical and antibiotic susceptibility profiles of *L*. *lactis*

The *L*. *lactis* strain was able to grow both at 30°C and 37°C. After 48 h of incubation the strain fermented ribose, galactose, glucose, fructose, mannose, N-acetylglucosamine, salicin, cellobiose, maltose, saccharose, trehalose and gentiobiose vigorously, and arbutin weakly.

The antibiotic susceptibility profile of *L*. *lactis* is shown in Table 2. Microbiological breakpoints for ampicillin, chloramphenicol, erythromycin, gentamicin, kanamicin, streptomycin, tetracycline and vancomycin reported by the FEEDAP document on the assessment of bacterial products used as feed additives in relation to antibiotic resistance [17] were used to categorize *L*. *lactis*. According to our results, the strain showed sensitivity to all the above antibiotics. We also tested additional drugs and identified the following MIC values: 64 μg/ml for amikacin, 4 μg/ml for ciprofloxacin, <1 μg/ml for clarithromycin, >8 μg/ml for linezolid and >16 μg/ml for rifampicin.

**Table 2:** Antibiotic susceptibility profile of *L*. *lactis* LMG 7930.

Antimicrobial agent	MIC range (μg/ml)	MIC^a^ (μg/ml)	EFSA cut-off value for resistance (μg/ml)
Amikacin	16–64	64	-
Ampicillin	1–16	<1	> 2
Ciprofloxacin	1–16	4	-
Clarithromycin	1–16	< 1	-
Chloramphenicol	1–16	8	> 8
Erythromycin	0.5–8	< 0.5	> 1
Gentamicin	4–32	<4	> 32
Kanamycin	4–64	<16	> 64
Linezolid	1–8	>8	-
Rifampicin	2–16	> 16	-
Streptomycin	16–64	32	> 32
Tetracycline	0.5–8	< 0.5	> 4
Vancomycin	0.5–8	< 0.5	> 4

^a^ MICs were established as the lowest antibiotic concentration that inhibited bacterial growth.

-, no drug cut-off value was defined by the FEEDAP Panel.

### Physiochemical properties of *L*. *lactis* cell surface

The adhesive characteristics of *L*. *lactis* in relation to xylene, chloroform and ethyl acetate were investigated. *L*. *lactis* exhibited a medium hydrophobicity (BATS xylene = 53.5%), low electron donor property (BATS chloroform = 14.9%) and no electron acceptor property.

**Fig 1:**
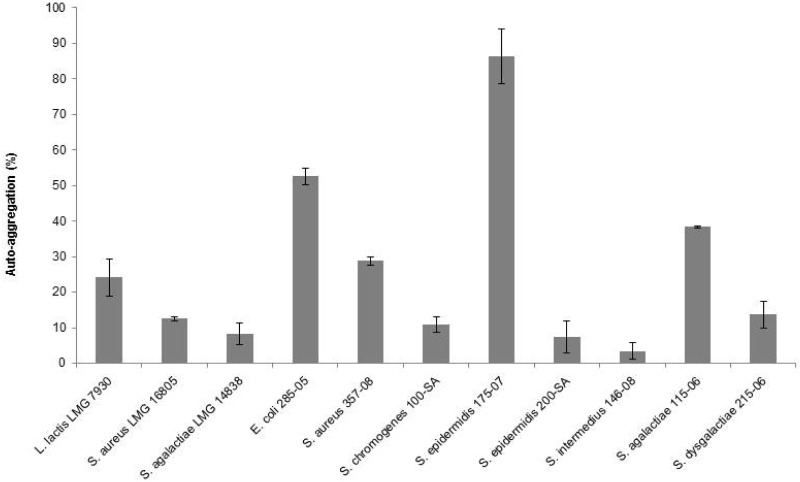
Auto-aggregation abilities of *L*. *lactis* LMG 7930 and mastitis-causing pathogens.

The auto-aggregation abilities of *L*. *lactis* and mastitis-causing pathogens are shown in Fig 1. Low auto-aggregative values were recorded for *L*. *lactis* (24.12%) and the majority of the pathogens (range from 3.43% to 28.77%). *E*. *coli* 285–05 and *S*. *agalactiae* 115–06 field isolates showed medium auto-aggregative abilities (52.52% and 38.33%, respectively); the pathogen *S*. *epidermidis* 175–07 demonstrated the greatest auto-aggregative capability (86.38%).

*L*. *lactis* did not show co-aggregative abilities in relation to any of the tested pathogens under our experimental conditions.

### *L*. *lactis* antagonistic activity

Results of the agar spot test are shown in Table 3. *L*. *lactis* inhibited the majority of the pathogens tested, with inhibition zone radii ranging from 1 to 3 mm. The greatest antimicrobial activity was recorded against the *S*. *agalactiae* 115–06 field isolate (halo radius of 3 mm) and the nisin-sensitive *L*. *cremoris* LMG 7951 (halo radius of 5 mm) used as a positive control. No inhibitory effect was observed towards *E*. *coli* 285–05, *S*. *intermedius* 146–08 and *S*. *dysgalactiae* 215–06.

**Table 3:** Antimicrobial activity of *L*. *lactis* LMG 7930 towards mastitis-causing pathogens.

Bacterial strain	Inhibition ± SD^a^
*S*. *aureus* LMG 16805	1 ± 0
*S*. *agalactiae* LMG 14838	1 ± 0
*E*. *coli* 285–05	-
*S*. *aureus* 357–08	1 ± 1
*S*. *chromogenes* 100-SA	2 ± 1
*S*. *epidermidis* 175–07	1 ± 1
*S*. *epidermidis* 200-SA	2 ± 0
*S*. *intermedius* 146–08	-
*S*. *agalactiae* 115–06	3 ± 2
*S*. *dysgalactiae* 215–06	-
*L*. *cremoris* LMG 7951	5 ± 1

^a^ The halo radii are expressed as the mean (mm) ± standard deviation of triplicate spots from two independent experiments;

-, no inhibition.

### Adhesion and invasion capabilities

**Fig 2:**
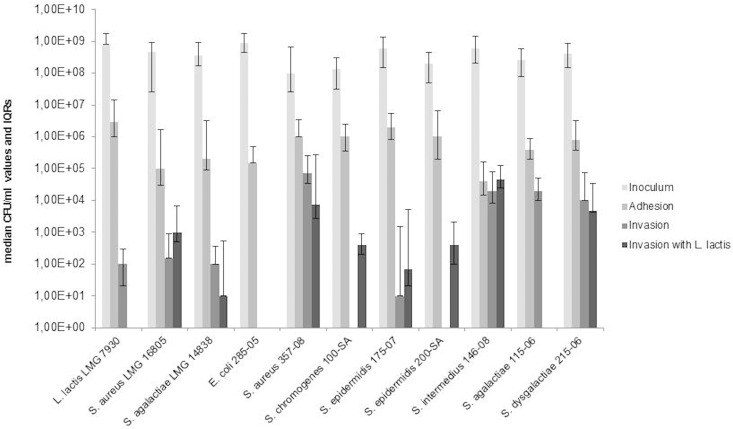
Adhesion and invasion capabilities. Inoculum levels, adhesion capabilities to and invasion abilities in BME-UV1 of *L*. *lactis* and mastitis-causing pathogens alone and in presence of *L*. *lactis* LMG 7930 after 2 h of incubation at 37°C are shown. Results are expressed as medians and interquartile ranges of CFU/ml values.

Median adhesion and invasion values of *L*. *lactis* and mastitis-causing pathogens are shown in Fig 2. Similar amounts of bacterial inoculum were applied to the tests (median values ranged from 1.0 x 10^8^ to 9.0 x 10^8^ CFU/ml). The most adhesive bacteria to BME-UV1 were *L*. *lactis* and the pathogenic field isolates *S*. *aureus* 357–08, *S*. *chromogenes* 100-SA, and both *S*. *epidermidis* strains (median values ranged from 1.0 x 10^6^ to 3.0 x 10^6^ CFU/ml), followed by *E*. *coli* 285–05, *S*. *agalactiae* 115–06, *S*. *dysgalactiae* 215–06 and the two reference mastitis-causing pathogens (median values ranged from 1.0 x 10^5^ to 8.0 x 10^5^ CFU/ml). The least adhesive was *S*. *intermedius* 146–08 (median value of 4.0 x 10^4^ CFU/ml).

Bacterial internalization capabilities in BME-UV1 were also different: *S*. *aureus* 357–08, *S*. *intermedius* 146–08, *S*. *agalactiae* 115–06 and *S*. *dysgalactiae* 215–06 field isolates were the most invasive (median values ranged from 1.0 x 10^4^ to 7.0 x 10^4^ CFU/ml). The reference *S*. *aureus* and *S*. *agalactiae* strains (median values of 1.0 x 10^2^ and 1.5 x 10^2^ CFU/ml, respectively) and *S*. *epidermidis* 175–07 (median value of 1.0 x 10^1^ CFU/ml) were the least invasive. It is noted, *L*. *lactis* exhibited an internalization capability, albeit modest (median value of 1.0 x 10^2^ CFU/ml). Conversely, *E*. coli 285–05, *S*. *chromogenes* 100-SA and *S*. *epidermidis* 200-SA showed no invasive abilities under our experimental conditions.

*L*. *lactis*, when simultaneously added with the pathogen to the cells, did not reduce statistically pathogen internalization. However a trend to reduce the median invasion values of *S*. *aureus* 357–08 (from 7.0 x 10^4^ to 7.5 x 10^3^ CFU/ml), *S*. *agalactiae* 115–06 (from 2.0 x 10^4^ to 0 CFU/ml) and *S*. *dysgalactiae* 215–06 (from 1.0 x 10^4^ to 4.70 x 10^3^ CFU/ml) field isolates, as well as that of *S*. *agalactiae* LMG 14838 reference (from 1.0 x 10^2^ to 1.0 x 10^1^ CFU/ml) was observed. The median invasion values of *S*. *epidermidis* 175–07 and *S*. *intermedius* 146–08 remained unchanged in the presence of *L*. *lactis*. Conversely, a two-log increase of the median invasion values of *S*. *chromogenes* 100-SA and *S*. *epidermidis* 200-SA (from 0 to 4.0 x 10^2^ CFU/ml) and an approximately one-log increase of the median invasion value of *S*. *aureus* LMG 16805 reference (from 1.5 x 10^2^ to 1.0 x 10^3^ CFU/ml) were observed in presence of *L*. *lactis*. Those rises however were not statistically significant.

## Discussion

One of the sustainable antibiotic alternatives recently proposed for the treatment and prevention of mastitis in ruminants is the use of LAB as mammary probiotics [6–9]. In this work we have investigated *in vitro* the probiotic potential of *L*. *lactis* LMG 7930 towards mastitis-causing pathogens in order to evaluate this strain as a potential candidate for use as an antibiotic alternative for treating mastitis in dairy ruminants.

*L*. *lactis* LMG 7930 is a commercially available nisin-producing strain. It is used in Swiss cheese manufacture to suppress gas production by Clostridia. It is considered safe for human consumption.

*L*. *lactis* subsp. *lactis* strains are widely used as starter cultures in the food industry for the production of cheeses and fermented milks. Because of their long-time use in various food and feed preparations, *L*. *lactis* strains are recognized food-grade organisms that do not pose a health risk for consumers or the environment. However, the antimicrobial susceptibility of food-associated LAB such as *L*. *lactis* strains should be determined to prevent potential antibiotic resistance traits from being transferred to human or animal commensal flora and to pathogenic bacteria. EFSA requires as part of its Qualified Presumption of Safety approach the safety assessment of bacteria deliberately introduced into the food chain, namely to ascertain that acquired resistance determinants to antimicrobials of clinical importance are absent. The cut-off values identified by the FEEDAP Panel [17] were here investigated to ensure the absence of acquired antimicrobial resistance properties in the *L*. *lactis* strain. We found that *L*. *lactis* LMG 7930 was sensitive towards antibiotics whose drug monitoring is recommended by EFSA. No genetic investigation to determine the presence of antibiotic resistance determinants was conducted, as suggested by the FEEDAP Panel for drug sensitive strains.

*L*. *lactis* has recently been isolated from bovine mastitis cases [21,22]. Plumed-Ferrer and colleagues [23], comparing the characteristics of *L*. *lactis* isolates from bovine mastitis with dairy starter strains, sought to find differences between pathogenic and non-pathogenic strains. No differences between them were found genotypically. However, authors described some phenotypic distinctions between mastitis isolates and typical starter strains, such as an improved carbohydrate fermentation capacity showed by the former, although no reliable safety criteria was provided [23]. To better characterize *L*. *lactis* LMG 7930 we determined its carbohydrate fermentation profile. Like bulk starter strains, the *L*. *lactis* strain was able to ferment galactose, glucose, fructose, mannose and N-acetylglucosamine. In addition, the strain fermented eight out of 14 carbohydrates metabolized by mastitis isolates studied by Plumed-Ferrer *et al*. [23]: arbutin, ribose, salicin, cellobiose, maltose, saccharose, trehalose and gentiobiose. These differences might not be considered as actual virulence factors: while on the one hand the improved carbohydrate fermentation capacity demonstrated by *L*. *lactis* compared to starter strains may offer the advantage of better surviving in the body of homeothermic animals just like pathogens, on the other this is also a typical property associated with potential probiotic strains.

We also investigated the antimicrobial activity of *L*. *lactis* against mastitis-causing pathogens. *L*. *lactis* exhibited antagonistic properties towards many of the pathogens tested, as proven by the agar spot test. The observed antimicrobial activity is likely to be mostly due to the production of nisin, a 34 amino acid peptide which exhibits a broad spectrum of inhibitory activity against several Gram-positive bacteria and usually no effect on Gram-negative bacteria, yeasts and molds [24]. Here *L*. *lactis* was in fact unable to inhibit the growth of *E*. *coli*. However, we cannot exclude the effect of other antimicrobial compounds or pH of culture.

*L*. *lactis* underwent physiochemical characterization of the cell surface. BATS tests, also called MATS (microbial adhesion to solvents) tests, are used to assess the hydrophobic/hydrophilic, electron donor (basic) and electron acceptor (acidic) characteristics of the bacterial surface, in order to predict the colonizing ability of a bacterial strain [25,26]. *L*. *lactis* exhibited medium surface hydrophobicity, low basic property and no electron acceptor capability. These results concur with literature, where probiotic strains showed nonacidic and poor electron acceptor properties [27].

Although several studies have reported that hydrophobicity, electron-donor/electron-acceptor properties play a crucial role in the microbial colonization, it is important to emphasize that these cell surface characteristics are not sufficient to explain these phenomena [28]. Physiochemical properties of the bacterial cell surface can in fact be modified depending on the physiological state of the cell, nutrient composition [29,30] and pH of the growth medium [28].

We also assessed the cell-binding properties of *L*. *lactis*, namely auto-aggregation and co-aggregation abilities. Both abilities are believed to be important features in the selection of a potential probiotic strain, because they may orchestrate the bacterial adhesion to epithelial cells (auto-aggregation) and prevent colonization by pathogenic microorganisms (co-aggregation) [31]. *L*. *lactis* showed low auto-aggregation and no co-aggregation abilities towards any of the tested pathogens. Literature shows a discrepancy in the auto-aggregation and co-aggregation abilities of LAB. In previous studies LAB isolated from milk exhibited either low [10] or no auto-aggregative phenotype [11]. In another study, on the other hand, most probiotic strains tested showed high auto-aggregation abilities and co-aggregation capabilities with foodborne pathogens [18]. Our results further support the strain dependence of the cell surface and cell-binding properties of a LAB.

Different studies have reported that bacterial adhesion to host cells is an initial step in bacterial infection and host colonization in different host niches [32,33]. Here we have assessed adhesion to and invasion into the bovine mammary epithelial cell line BME-UV1of *L*. *lactis*, as well as its competitive inhibition capability against pathogen invasion. *L*. *lactis* was one of the most adhesive to BME-UV1 among tested bacteria, but its internalisation was low (median value of 1.0 x 10^2^ CFU/ml). Previous studies have documented the internalization capability of LAB into MAC-T cells, which was generally lower than that of mastitis-causing pathogens [12,15]. The low internalization capability of LAB species can be considered as an advantage, since it considerably limits the risk of tissue invasion by the potential probiotic candidate.

*L*. *lactis* was unable to reduce significantly pathogen invasion; however, a trend to decrease the median internalisation values of *S*. *aureus* 357–08, *S*. *agalactiae* 115–06 and *S*. *dysgalactiae* 215–06 field isolates, as well as that of *S*. *agalactiae* LMG 14838 reference, was observed. The ability of LAB to inhibit the internalization capability of *S*. *aureus* has already been reported in literature [12,15]. We also documented the ability of *L*. *lactis* to increase the median internalization values of some pathogens, such as *S*. *chromogenes* 100-SA, *S*. *epidermidis* 200-SA and *S*. *aureus* LMG 16805 reference, even though these increases were not statistically significant. This phenomenon has not previously been documented in literature.

In conclusion, we showed that *L*. *lactis* LMG 7930 was sensitive to tested drugs. It was also able to contrast the growth of many of the pathogens tested by means of the production of antagonistic substances. In addition, the strain adhered to bovine mammary epithelial cells. Although the strain did not reduce statistically pathogen internalization, a trend to reduce the median internalization values of many pathogens was observed. Our findings suggest that this strain might be a promising candidate for the development of new strategies of mastitis control in ruminants. The next mandatory step will be to assess this strain *in vivo* for safety and efficacy under field conditions.
